# Targeted degradation of α-synuclein by arginine-based PROTACs

**DOI:** 10.1016/j.jbc.2025.110449

**Published:** 2025-07-02

**Authors:** Linjing Shen, Jianchao Zhang, Zhaoran Wang, Yaxuan Liu, Shengjin Cui, Hai Rao

**Affiliations:** 1 Department of Biochemistry, SUSTech Homeostatic Medicine Institute, School of Medicine, Southern University of Science and Technology, Shenzhen, China; 2 Clinical Laboratory, The University of Hong Kong-Shenzhen Hospital, Shenzhen, China; 3 Key University Laboratory of Metabolism and Health of Guangdong, Southern University of Science and Technology, Shenzhen, China

**Keywords:** PROTAC, N-end rule, amino acid, arginine, α-synuclein, Parkinson's disease, AATac

## Abstract

Parkinson's disease (PD), the second most prevalent neurodegenerative disorder, is associated with α-synuclein (α-syn) overexpression or mutation, leading to harmful aggregates and neuronal apoptosis. Effective drugs that inhibit or reduce α-syn accumulation remain challenging. Targeted protein degradation (TPD) technology offers a novel solution by utilizing the ubiquitin-proteasome pathway to target specific proteins for destruction. Here, we have developed Proteolysis Targeting Chimera (PROTAC) to target α-syn for degradation. Specifically, our PROTACs employ the amino acid arginine (Arg) as the E3 ligase ligand and a benzothiazole-aniline variant as the warhead for α-syn. The efficacy of these PROTACs in degrading α-syn and its aggregates was tested in mammalian cells and *Caenorhabditis elegans* (*C*. *elegans*) models. Arg-PEG1-T^**α-syn**^ shows the highest degradation effect in mammalian cells for both wild-type α-syn and the α-syn (A53T) mutant. UBR1 is the ubiquitin E3 ligase responsible for PROTAC-mediated degradation. Furthermore, Arg-PEG1-T**^α-syn^** significantly reduces α-syn aggregates and associated toxicities in both mammalian cells and *C*. *elegans*. These findings highlight the potential of a single amino acid-based PROTAC targeting α-syn for degradation, representing a possible therapeutic approach for PD and other synucleinopathies.

The rising prevalence of age-related neurodegenerative disorders including Alzheimer's disease (AD) and Parkinson's disease (PD) poses a significant societal and economic challenges. These neurodegenerative diseases are marked by progressive neurodegeneration and neuronal loss, and are characterized by misfolded protein aggregates including β-amyloid (Aβ), tau, α-synuclein (α-syn), and TDP-43 (1, 2, 3, 4, 5). The treatments for these diseases are primarily limited to symptomatic relief (6, 7, 8). PD is the second most common neurodegenerative disease after AD. The protein α-syn was identified decades ago as a major pathogenic factor in PD (9, 10, 11, 12). Overexpression of α-syn wild type (WT) or its A53T mutant can cause its misfolding, oligomerization, and aggregation. The α-syn oligomers and aggregations are major constituents of the Lewy bodies in PD (10), which are neurotoxic and ultimately lead to the neuron apoptosis (12, 13, 14). Therefore, reducing α-syn accumulation is a promising therapeutic approach against PD. Nevertheless, despite enormous efforts from academy and biotech industry, there is no cure for PD currently.

Proteolysis Targeting Chimera (PROTAC) technology offers a novel solution by leveraging the ubiquitin-proteasome system to degrade target proteins (15, 16, 17). PROTAC molecules typically consist of three components: a warhead binding to the protein of interest (POI), a ligand recognized by an E3 ubiquitin ligase, and a linker connecting the two (Fig. 1*A*). This system induces target ubiquitylation and subsequent degradation without requiring direct inhibition of its activity. In recent years, PROTAC has gained significant attention in the pharmaceutical industry for its potential to treat various diseases, including cancers, viral or bacterial infections, and neurodegenerative diseases (18, 19, 20, 21, 22, 23, 24, 25, 26).

**Figure 1:**
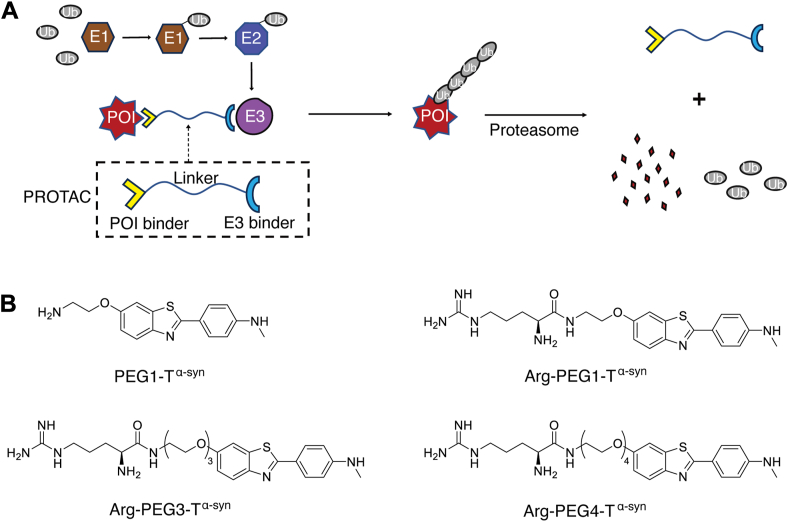
**PROTAC designs**. *A*, the PROTAC (proteolysis-targeting chimera) functions by simultaneously binding to the protein of interest (POI) and the E3 ligase through its dual ligands. These interactions facilitate the ubiquitiylation of the POI, leading to its subsequent degradation by the proteasome. *B*, the chemical structure of the PROTAC candidates in this study consists of three main components: an arginine (Arg) ligand targeting the E3 ligase, a benzothiazole-aniline (BTA) ligand targeting α-synuclein (T^α-syn^), and a linker composed of varying numbers of polyethylene glycol (PEG) units connecting the two ligands. The candidates are designated as Arg-PEG1-T^α-syn^, Arg-PEG3-T^α-syn^ and Arg-PEG4-T^α-syn^, based on the number of PEG units. A negative control, PEG1-T^α-syn^, consisting of BTA with only one PEG unit, was also included in the study.

Most of the PROTACs designed have employed the ligands for either VHL or CRBN E3, which have been adapted for α-syn degradation as well (18, 24, 25, 26, 27, 28). We have developed a set of single amino acid-based PROTACs (termed AATacs) to target various oncogenic drivers (*e*.*g*., BCR-ABL, EML4-ALK, ERRα) for proteasome-mediated destruction in cells and/or xenograft mice (29, 30, 31). A destabilizing residue (*e*.*g*., Arg, Leu, Lys) exposed at the N-terminus of a protein can be recognized by a ubiquitin E3 ligase (*e*.*g*., UBR1, UBR2, UBR4, or UBR5), triggering substrate ubiquitylation and degradation (32, 33). The dissection of the link between the N-terminal residue and the half-life of a protein in eukaryotes led to the first genetically defined ubiquitin-proteasome mediated degradation route termed the N-end rule pathway (32, 33). The single amino acid-based degradation signal offers several distinct advantages over other degrons, including smaller size, naturally existing without toxicity, recognized by highly conserved E3s. Here, we have extended the application of AATacs to degrade α-syn, a culprit of PD, using Arg as the E3 ligand. The efficacy of these AATacs in degrading α-syn was evaluated in mammalian cells and *Caenorhabditis elegans* (*C*. *elegans*) models. Our study brings a new PROTAC approach for PD and other synucleinopathies.

## Results

### Design and synthesis of the PROTACs targeting **α**-syn

As we have previously developed N-end rule-based PROTACs for cancer treatment (29, 30, 31), we explored whether this class of PROTACs could also be applied to the treatment of neurodegenerative diseases. Since excess levels of α-syn, whether wild-type or mutant, have been strongly implicated in the pathogenesis of PD (12, 13, 34), we selected α-syn as the target protein for our investigation into PD. To design our PROTAC, we utilized an Arg residue as the E3 ligase-binding ligand to recruit UBR family E3 ligases (Fig. 1*B*). Since previous studies demonstrated the strong affinity of a benzothiazole-aniline (BTA), a variant derived from the amyloid stain dye Thioflavin T, to cross β-sheet structures found in aggregates associated with α-syn (35, 36, 37), we chose BTA as the warhead targeting α-syn (Fig. 1*B*).

The composition and length of the linker are critical determinants of PROTAC efficacy. Polyethylene glycol (PEG) is often employed as a linker due to its excellent solubility, cell permeability, and flexible length (38). With these considerations, we incorporated varying numbers of ethylene glycol units as the linker between Arg and BTA for the α-syn-targeting PROTACs (Fig. 1*B*).

### PROTACs facilitate the degradation of **α**-syn in mammalian cells

**Figure 2:**
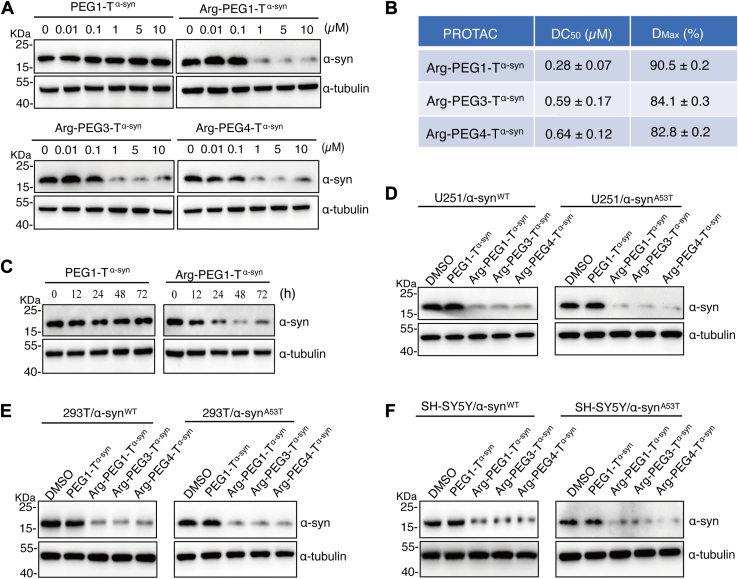
**Arg-PEG (1,3,4)-T^α-syn^ promoted α-syn ^WT/A53T^ degradation in mammalian cell lines**. *A*, immunoblot analysis of U251 cells overexpressing α-syn^A53T^ (U251/α-syn^A53T^) treated with PROTACs Arg-PEG(1,3,4)-T^α-syn^ at varying concentrations for 48 h. *B*, summary of DC_50_ (the PROTAC concentration required to induce 50% degradation of the target protein) and D_Max_ (the maximum degradation percentage of the target protein achieved with 5 μM PROTAC) for α-syn^A53T^ degradation by the PROTACs. *C*, immunoblot analysis of U251/α-syn^A53T^ cells treated with the negative control PEG-T^α-syn^ and the PROTAC Arg-PEG1-T^α-syn^ for different durations. *D*, immunoblot analysis of U251 cells overexpressing α-syn^WT^ (U251/α-syn^WT^) and U251/α-syn^A53T^ treated with PROTACs Arg-PEG(1,3,4)-T^α-syn^ at 1 μM for 48 h. *E*, immunoblot analysis of 293T cells overexpressing α-syn^WT^ (293T/α-syn^WT^) and α-syn^A53T^ (293T/α-syn^A53T^) treated with PROTACs Arg-PEG(1,3,4)-T^α-syn^ at 1 μM for 48 h. *F*, immunoblot analysis of SH-SY5Y cells overexpressing α-syn^WT^ (SH-SY5Y/α-syn^WT^) and α-syn^A53T^ (SH-SY5Y/α-syn^A53T^) treated with PROTACs Arg-PEG(1,3,4)-T^α-syn^ at 1 μM for 48 h.

The α-syn A53 T mutant (α-syn^A53T^), bearing one of the pathogenic mutations identified in rare familial PD, exhibits greater toxicity than wild-type α-syn (α-syn^WT^) due to its stronger propensity to aggregate (39, 40, 41). To evaluate the ability of the PROTACs to induce α-syn degradation, we first employed human glioblastoma U251 cells stably overexpressing α-syn^A53T^ *via* lentivirus infection. Compared to the negative control PEG1-T^α-syn^, all three PROTACs (*i*.*e*., Arg-PEG1-T^α-syn^, Arg-PEG3-T^α-syn^ and Arg-PEG4-T^α-syn^) significantly promoted the reduction of α-syn^A53T^ at 1 μM upon 48 h treatment (Fig. 2*A*). We further calculated the DC_50_ (the concentration required to induce 50% target reduction) and D_Max_ (the maximum reduction percentage achieved at 5 μM PROTAC) for α-syn^A53T^ degradation (Fig. 2*B*). Among the three PROTACs tested, Arg-PEG1-T^α-syn^ appeared to be the most potent with DC_50_ of 0.28 ± 0.07 μM and induced approximately 90.5% degradation of α-syn^A53T^.

We also investigated the time course of α-syn^A53T^ degradation induced by Arg-PEG1-T^α-syn^. Our analysis revealed that degradation could be detected as early as 12 h after treatment with 1 μM Arg-PEG1-T^α-syn^, reaching its peak at 48 h (Fig. 2*C*). Based on these findings, a 48-h treatment duration was adopted for most of the experiments described in this study.

We assessed the PROTAC-induced reduction of both α-syn^WT^ and α-syn^A53T^ in multiple cell lines (Fig. 2, *D–F*) (42). Compared to the negative controls (*i*.*e*., DMSO or PEG1-T^α-syn^), the three PROTACs with varying PEG linker lengths triggered efficient reduction of α-syn^WT^ or α-syn^A53T^ in three different mammalian cells (*i*.*e*., U251, 293T, SH-SY5Y) (Fig. 2, *D–F*). Collectively, these findings indicate that the PROTACs designed exhibit consistent degradation effects on α-syn.

### UBR1 E3 is involved in the regulation of **α**-syn^WT/A53T^ mediated by Arg-PEG1-T**^α-syn^**

**Figure 3:**
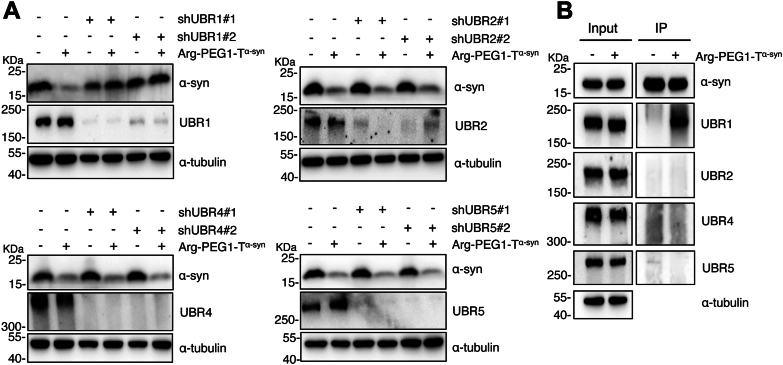
**Arg-PEG1-T^α-syn^ promoted α-syn^A53T^ degradation probably *via* ubiquitin ligase UBR1.***A*, immunoblot analysis of U251/α-syn^A53T^ cells stably expressing shRNA targeting UBR1, UBR2, UBR4, or UBR5, treated with either DMSO or 1 μM Arg-PEG1-T^α-syn^ for 48 h. *B*, immunoprecipitation of α-syn and its potential E3 ligases in U251/α-syn^A53^^T^ cells treated with 2 μM MG132, either in the presence or absence of Arg-PEG1-T^α-syn^, for 24 h.

The PROTAC-induced α-syn^A53T^ reduction is proteasome-dependent as it is reversed by the proteasome inhibitor MG132, but not the autophagy inhibitor CQ (Fig. S1). To identify the E3 ligase involved in the degradation of α-syn^A53T^ induced by Arg-PEG1-T^α-syn^, we knocked down UBR1, UBR2, UBR4, and UBR5 separately using shRNA in U251 cells expressing α-syn^A53T^ cells (Fig. 3). As illustrated in Figure 3*A*, the reduction of α-syn^A53T^ induced by Arg-PEG1-T^α-syn^ was markedly suppressed by UBR1 knockdown, whereas the knockdown of UBR2, UBR4, or UBR5 had little effect. Similarly, in U251 cells bearing α-syn^WT^, the reduction of α-syn^WT^ induced by Arg-PEG1-T^α-syn^ was also significantly impeded by UBR1 knockdown (Fig. S2).

As a E3 ubiquitin ligase is the substrate recognition component, we evaluated the interaction between UBR1 and α-syn^A53T^. We performed the immunoprecipitation assays to detect the association between α-syn^A53T^ and the N-end rule E3s, UBR1, UBR2, UBR4, and UBR5 in α-syn^A53T^ expressing cells treated with either 1 μM Arg-PEG1-T^α-syn^ or DMSO (Fig. 3*B*). We observed that UBR1 could be immunoprecipitated by α-syn in the presence of Arg-PEG1-T^α-syn^ treatment. Combined, these findings suggest that UBR1 is the E3 ubiquitin ligase involved in the degradation of α-syn induced by Arg-PEG1-T^α-syn^.

### Arg-PEG1-T^**α-syn**^ protects mammalian cells from the toxicity of human **α**-syn^WT/A53T^ overexpression

**Figure 4:**
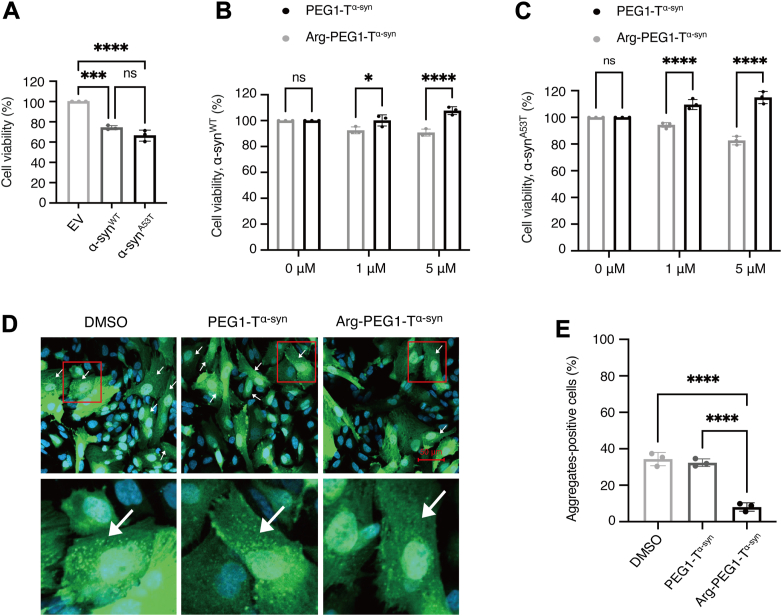
**Cell viability and α-syn aggregation in mammalian cells with Arg-PEG1-T^α–syn^ and negative control PEG1-T^α–syn^ treatment**. *A*, cell viability of SH-SY5Y cells harboring an empty vector (EV) or stably overexpressing human α-syn wild type (α-syn^WT^) or the A53T mutant (α-syn^A53T^) *via* lentivirus infection, without any treatment. One-way ANOVA with Tukey's multiple comparisons test was used. F = 82.33, *p* < 0.0001. ∗∗∗*p* < 0.001, ∗∗∗∗*p* < 0.0001, ns: *p* > 0.05. All individual data points super-imposed on the bar graphs were independent replicates. *B–C*, cell viability of SH-SY5Y cells stably overexpressing α-syn^WT^ or α-syn^A53T^ treated with varying concentrations of PEG1-T^α–syn^ and Arg-PEG1-T^α–syn^. Two-way ANOVA with Sidak's multiple comparisons test was used. ∗*p* < 0.05, ∗∗∗∗*p* < 0.0001. For *B*, interaction, F(2, 12) = 15.28, *p* = 0.0005; row factor, F(2, 12) = 3.214, *p* = 0.0762; column factor, F(1, 12) = 42.81, *p* < 0.0001. For *C*, interaction, F(2, 12) = 48.29, *p* < 0.0001; row factor, F(2, 12) = 1.900, *p* = 0.1920; column factor, F(1, 12) = 139.2, *p* < 0.0001. All individual data points super-imposed on the bar graphs were derived from independent experiments. *D*, fluorescence microscope images showing α-synuclein aggregates in SH-SY5Y cells stably overexpressing α-syn^A53T^ fused with GFP, achieved through lentivirus infection. The cells were treated with 5 μM of the respective compounds. *E*, statistical analysis of SH-SY5Y cells with α-syn^A53T^ aggregates. The percentage was calculated as the ratio of cells with green aggregates relative to the total number of GFP-positive cells (with or without aggregates). One-way ANOVA with Tukey's multiple comparisons test was used. F = 85.57, *p* < 0.0001. ∗∗∗∗*p* < 0.0001. All individual data points super-imposed on the bar graphs were independent replicates.

To investigate whether the PROTAC could mitigate the toxicity induced by α-syn^WT^ or α-syn^A53T^ overexpression, we carried out CCK8 assays in cells harboring an empty vector (EV), or stably overexpressing α-syn^WT^ or α-syn^A53T^ *via* lentivirus infections (Fig. 4). In the absence of drug treatment, overexpression of either α-syn^WT^ or α-syn^A53T^ significantly reduced cell viability (Fig. 4*A*), indicating that overexpression of both α-syn^WT^ and α-syn^A53T^ induced substantial cellular toxicity.

We treated the cells overexpressing α-syn^WT^ or α-syn^A53T^ with varying concentrations of PEG1-T^α–syn^ and Arg-PEG1-T^α–syn^ (Fig. 4, *B–C*). Compared to the negative control (DMSO, 0 μM) or PEG1-T^α–syn^ treatment, the PROTAC Arg-PEG1-T^α–syn^ significantly enhanced cell viability in α-syn^WT^ cells at 5 μM (Fig. 4*B*), and in α-syn^A53T^ cells starting at 1 μM (Fig. 4*C*). These results suggest that Arg-PEG1-T^α–syn^ could effectively protect cells from the toxicity induced by α-syn^WT^ or α-syn^A53T^ overexpression.

To determine whether the PROTAC could reduce α-syn aggregation in mammalian cells, we focused on α-syn^A53T^ overexpression, which is known to induce more pronounced aggregation than α-syn^WT^ (39, 40, 41). The mammalian cells bearing the α-syn^A53T^-GFP fusion were treated with DMSO, PEG1-T^α-syn^ control, or Arg-PEG1-T^α-syn^ for 48 h. Cells were then fixed with paraformaldehyde, permeabilized with Triton X-100, and mounted with antifade-mounting medium containing DAPI. Confocal microscopy revealed numerous green aggregates in cells expressing α-syn^A53T^ fused with GFP (Fig. 4*D*). Importantly, the treatment with Arg-PEG1-T^α-syn^ significantly reduced α-syn^A53T^ aggregates compared to DMSO or PEG1-T^α-syn^ treatment (Fig. 4*D*), whereas no green aggregates were observed in cells expressing α-syn^A53T^ and GFP separately (Fig. S3). Statistical analysis of α-syn^A53T^ aggregates (Fig. 4*E*), calculated as the percentage of cells with green aggregates relative to the total number of GFP-positive cells, demonstrated that the Arg-PEG1-T^α-syn^ treatment reduced α-syn^A53T^ aggregates by nearly two-thirds compared to the negative control groups (DMSO or PEG1-T^α-syn^). These findings indicate that Arg-PEG1-T^α-syn^ effectively reduces α-syn^A53T^ aggregation in mammalian cells.

### The PROTAC Arg-PEG1-T**^α-syn^** decreases **α**-syn aggregates and improves the dopaminergic neuronal impairment and the locomotion of *C*. *elegans*

The hallmark features of PD include the presence of Lewy bodies containing α-syn aggregates in dopaminergic neurons, along with dopaminergic neuronal degeneration and movement disorders. In addition to cell-based studies above, we further investigated the effects of the Arg-PEG1-T^α-syn^ on α-syn aggregate clearance, neuronal impairment, and locomotion in the animal model *C*. *elegans*. *C*. *elegans* offers unique advantages for our analysis, including its transparent body that facilitates *in vivo* observation of α-syn aggregates and neuronal impairment, its slow and simple bending movements that are ideal for behavioral analysis under a microscope, and its moderate growth rate and lifespan that allow for timely phenotypic observations following treatment.

**Figure 5:**
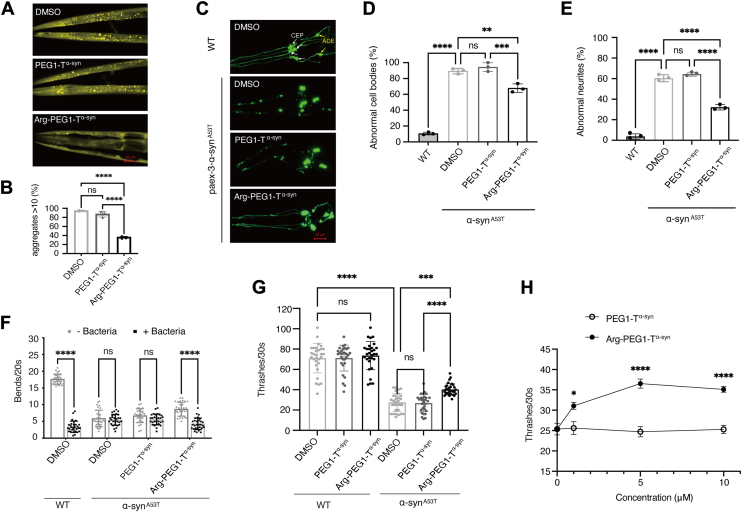
**Dopaminergic neuronal impairment, behavior, and α-syn aggregation in *C*. *elegans* with Arg-PEG1-T^α–syn^ and negative control PEG1-T^α–syn^ treatment**. *A–B*, microscopic observation, and statistical analysis of α-syn aggregates in the muscular cells of the head region in day-5 adults. Images were captured at 400 × magnification. The NL5901 strain, which exhibits pan-muscular overexpression of human α-syn^WT^ fused with YFP and displays α-syn aggregates in muscles, was used for this assay. Worms with more than 10 aggregates were counted. For *B*, One-way ANOVA with Tukey's multiple comparisons test was used. F = 191.2, *p* < 0.0001. ∗∗∗∗*p* < 0.0001, ns: *p* > 0.05. All individual data points super-imposed on the bar graph were derived from independent experiments. *C–E*, microscopic observation, and statistical analysis of dopaminergic neuron impairments in the head region of day-5 adults. The wild type (WT) strain, with normal dopaminergic neurons marked by pan-dopaminergic neuronal GFP overexpression, served as the negative control. The α-syn^A53T^ strain, characterized by pan-neuronal overexpression of human α-syn^A53T^ and pan-dopaminergic neuronal GFP overexpression, exhibits impaired dopaminergic neurons and was used for the test. Images were obtained at 400 × magnification. CEP: cephalic neurons; ADE: anterior deirid neurons. For statistical analysis in panels *D–E*, the number of worms with degenerated cell bodies or broken synapses was counted, and their percentage relative to the total number of worms was calculated. One-way ANOVA with Tukey's multiple comparisons test was used. ∗∗*p* < 0.01, ∗∗∗*p* < 0.001. ∗∗∗∗*p* < 0.0001, ns: *p* > 0.05. For *D*, F = 240.0, *p* < 0.0001. For *E*, F = 322.0, *p* < 0.0001. All individual data points super-imposed on the bar graph were independent replicates. *F*, basal slowing response assay for day-5 adults, used to assess dopaminergic neuron function. Worms with normal dopaminergic neurons slow their movement in the presence of bacteria, while those with impaired dopaminergic neurons show no such behavioral change. The WT strain and the α-syn^A53T^ strain with impaired dopaminergic neurons were used for this test. Two-way ANOVA with Sidak's multiple comparisons test was used. Interaction, F(3, 224) = 168.8, *p* < 0.0001; row factor, F(3, 224) = 83.38, *p* < 0.0001; column factor, F(1, 224) = 423.2, *p* < 0.0001. ∗∗∗∗*p* < 0.0001, ns: *p* > 0.05. All individual data points super-imposed on the bar graph were independent biological replicates. *G*, thrashing assay (counting the bends of worms in M9 buffer to assess locomotion) for day-5 adults treated with 5 μM of compounds. The WT strain and the α-syn^A53T^ strain were used for this test. One-way ANOVA with Tukey's multiple comparisons test was used. F = 123.6, *p* < 0.0001. ∗∗∗*p* < 0.001, ∗∗∗∗*p* < 0.0001, ns: *p* > 0.05. All individual data points super-imposed on the bar graph were independent biological replicates. *H*, thrashing assay for day-5 adults of α-syn^A53T^ strain treated with varying concentrations of compounds. Two-way ANOVA with Sidak's multiple comparisons test was used. Interaction, F(3, 181) = 9.620, *p* < 0.0001; row factor, F(3, 181) = 8.022, *p* < 0.0001; column factor, F(1, 181) = 68.65, *p* < 0.0001. Significant differences from PEG1-T^α-syn^ treatment group, ∗*p* < 0.05, ∗∗∗∗*p* < 0.0001, ns: *p* > 0.05.

We first utilized *C*. *elegans* strain NL5901 (P_*unc-54*_::α-syn^WT^::YFP, obtained from the *Caenorhabditis* Genetics Center) to observe α-syn aggregates. This strain exhibits pan-muscular overexpression of human α-syn^WT^ fused with YFP and has been widely used to study modifiers of α-syn aggregation (43, 44, 45, 46, 47, 48). As shown in Figure 5*A*, worms treated with DMSO or PEG1-T^α-syn^ displayed numerous bright yellow fluorescent aggregates in muscle cells. In contrast, worms treated with Arg-PEG1-T^α-syn^ showed a significant reduction in α-syn aggregates. For quantitative statistical analysis, we counted the number of worms with more than 10 aggregate-like dots and calculated their percentage relative to the total number of worms (Fig. 5*B*). While over 80% of DMSO- or control PEG1-T^α-syn^-treated worms exhibited more than 10 aggregate dots, only about 30% of Arg-PEG1-T^α-syn^-treated worms showed a similar level of aggregation (Fig. 5*B*). These results are consistent with our earlier cell-based findings (Fig. 4, *E–F*).

In a previous study, pan-neuronal overexpression of α-syn^WT^ or α-syn^A53T^ in *C*. *elegans* significantly induced dopaminergic neuronal loss and motor deficits (49). However, over time, the *C*. *elegans* strain with pan-neuronal overexpression of α-syn^WT^ lost these phenotypes. likely due to the failure of exogenous gene integration into the genome, resulting in gene loss during continuous passaging. This issue did not occur in strains overexpressing α-syn^A53T^ pan-neuronally (50). Therefore, we selected the α-syn^A53T^ pan-neuronal overexpression strain to evaluate the effect of Arg-PEG1-T^α-syn^ on neuronal impairment and locomotion defects. This strain (UM0020, P_*unc-119*_::YFP, P_*unc-119*_::sid-1, P_*dat-1*_::GFP, P_*aex-3*_:: α-syn^A53T^, obtained from the Garry lab) (50) also expresses GFP in dopaminergic neurons, enabling visualization under a microscope. Wild type (WT) *C*. *elegans* with normal dopaminergic neurons marked by GFP served as a negative control. Since worms older than 4 days exhibit similar neuronal loss (49), we focused on day-5 adults for our observations. Using confocal microscopy, we examined six dopaminergic neurons in the head of *C*. *elegans*, including four CEPs (cephalic neurons) and two ADEs (anterior deirid neurons). Intact dopaminergic neurons were observed in the WT strain, while the UM0020 strain showed degenerated cell bodies and broken synapses (Fig. 5*C*). Compared to the DMSO control and PEG1-T^α-syn^-treated groups, which exhibited significant neuronal degeneration and synaptic breakage, the Arg-PEG1-T^α-syn^-treated group displayed improved cell bodies and synapses (Fig. 5*C*). Statistical analysis based on Figure 5*C* revealed that Arg-PEG1-T^α-syn^ treatment significantly reduced degenerated cell bodies and broken synapses, with synaptic breakage decreasing by nearly half compared to PEG1-T^α-syn^ treatment (Fig. 5, *D–E*).

To assess dopaminergic neuron function, we performed a basal slowing response assay, which measures the ability of worms to slow their movement in the presence of bacteria—a behavior dependent on intact dopaminergic neurons. Synchronized day-5 adults of the WT and UM0020 strains were used for this assay, with drug treatments initiated at the L1 stage. As shown in Figure 5*F*, DMSO-treated WT worms significantly slowed their movement upon encountering bacteria. In contrast, DMSO- or PEG1-T^α-syn^-treated UM0020 worms showed no overt behavioral change. However, Arg-PEG1-T^α-syn^-treated UM0020 worms exhibited a significant improvement in this phenotype, although their locomotion capacity remained weaker than that of WT worms (Fig. 5*F*).

In addition to the basal slowing response assay, we assessed locomotor function by monitoring thrashing behavior (body bends in M9 buffer) of *C*. *elegans*. WT and UM0020 strains were treated with 5 μM Arg-PEG1-T^α-syn^ or PEG1-T^α-syn^ for varying durations to evaluate potential compound toxicity (Figs. 5*G*, S3). The WT strain served as a control. No significant differences in thrashing activity were observed between Arg-PEG1-T^α-syn^-treated and DMSO- or PEG1-T^α-syn^-treated WT worms at either day-1 (Fig. S4) or day-5 (Fig. 5*G*) of adulthood, indicating minimal toxicity. UM0020 worms exhibited significantly reduced movement compared to WT worms due to the toxicity of pan-neuronal α-syn^A53T^ overexpression. However, treatment with Arg-PEG1-T^α-syn^ from the L1 stage to day-1 (Fig. S4) or day-5 (Fig. 5*G*) adulthood significantly rescued locomotor deficits in UM0020 worms compared to both DMSO- and PEG1-T^α-syn^-treated groups. This therapeutic effect was more pronounced in day-5 adults (Fig. 5*G*). Further analysis demonstrated that Arg-PEG1-T^α-syn^ significantly improved locomotor performance in UM0020 worms compared to PEG1-T^α-syn^-treated controls starting at 1 μM (Fig. 5*H*). In summary, Arg-PEG1-T^α-syn^ effectively improved dopaminergic neuron function and locomotion in *C*. *elegans* with pan-neuronal overexpression of human α-syn^A53T^, highlighting its potential therapeutic relevance.

## Discussion

As an emerging therapeutic approach with distinct advantages, PROTAC has garnered significant interests in academy and the pharmaceutical industry. A variety of PROTAC molecules have been developed to degrade many target proteins, showcasing potential in treating a wide array of diseases including cancer, viral infections, immune disorders, and neurodegenerative diseases (19, 20, 21, 22, 23, 51). While the majority of the PROTAC field employed the ligands for CRBN or VHL E3, we have adapted single amino acids as the E3 ligand with unique advantages (29, 30) including the following (1): They possess a smaller molecular weight, enhancing cell permeability (2); they attract a broader range of potent E3 ligases, including UBR1, UBR2, UBR4, and UBR5 (3); and the degradation rate can be modulated by altering the amino acid. While previous studies primarily focused on oncology-related proteins (29, 30, 31), this study reveals that single amino acid–based PROTACs (*i*.*e*., AATacs) can also be applied to neurodegenerative disease, such as PD, by targeting α-syn degradation.

In our exploration of the N-end rule–based PROTACs for PD treatment, we have selected the amino acid Arg as the degradation signal, which was previously employed in the PROTACs against various cancer drivers (*e*.*g*., ERRα, BCR-ABL, *etc*.). UBR1, UBR2, UBR4, and UBR5 are the potential E3 ubiquitin ligases that recognize the N-terminal Arg. Interestingly, whereas UBR1 appears to be the E3 mainly responsible for Arg-PEG1-Tα-syn -induced α-syn reduction (Fig. 3), UBR4 E3 seems to play a more significant role with redundant contributions from UBR1 and UBR2 E3 in Arg-based PROTAC (*i*.*e*., Arg-PEG1-Dasa) mediated BCR–ABL degradation in K562 cells (30). The distinct employment of E3s could be due to differences in cell types and/or E3 expression or the efficacy in the functional assembly of the target-PROTAC-E3 complex.

The warhead for α-syn is a BTA variant derived from the amyloid stain dye ThT, which has a high binding affinity for cross β-sheet structures (35, 36). This biochemical property of BTA allows its application not only to α-syn but also to other proteins prone to forming cross β-sheet structures, such as tau, Aβ, and TDP-43, as evidenced by several studies (35, 36, 37). For example, in a previous study, PROTAC 2 uses the BTA variant to bring the TAR DNA-binding protein (TDP-43) to the E3 CRBN *via* pomalidomide for degradation (37). It is well-established that different neurodegenerative diseases are characterized by distinct toxic proteins with β-sheet structures localized in various cell types and tissues. For instance, toxic α-syn primarily affects dopaminergic neurons in PD, tau and Aβ aggregates are predominantly found in the cerebrum in AD, and TDP-43 aggregates are implicated in motor neurons in ALS. These toxic proteins can also spread to other cell types and tissues as the disease progress (52, 53, 54, 55, 56, 57, 58, 59). Therefore, our amino acid-based PROTACs may not only be effective for PD by targeting α-syn degradation but could also be adapted toward other neurodegenerative diseases, including AD by targeting tau and Aβ degradation, and ALS by targeting TDP-43 degradation.

To our knowledge, this is the first demonstration of an N-end rule–based PROTAC (*i*.*e*., Arg-PEG1-T^α-syn^) effectively targets a key factor in the neurodegenerative disorder, α-syn, reduces α-syn aggregates, mitigates related toxicities and synucleinopathies. Given the distinct advantages associated with single amino acid-based degradation signals, it will be important to optimize and improve the application of AATacs in various age-related neurodegenerative diseases.

## Experimental procedures

### Reagents

Antibodies used include anti-α-syn (catalog no.: ab138501; 1:3000 dilution) from Abcam; anti-α-tubulin (Cat. No.: HRP-66031, 1:5000 dilution) from Proteintech; anti-UBR1 (Cat. No.: sc-515753, 1:1000 dilution) from Santa Cruz Biotechnology; anti-UBR2 (Cat. No.: ab217069, 1:1000 dilution) from Abcam; anti-UBR4 (Cat. No.: ab86738, 1:1000 dilution) from Abcam; anti-UBR5 (Cat. No.: 65,344, 1:1000 dilution) from Cell Signaling Technology. Antibody specificity was confirmed by target protein knockdown. Radioimmunoprecipitation (RIPA) lysis buffer was prepared with 50 mM Tris-HCl [pH 8.0], 1% NP-40, 150 mM NaCl, 0.1% SDS, 0.5% sodium deoxycholate. 10 × TBST buffer was prepared with 100 mM Tris-HCl, 150 mM NaCl, and 0.5% Tween 20 at pH 7.5. M9 buffer was prepared with 3 g KH_2_PO_4_, 6 g Na_2_HPO_4_, 5 g NaCl, 1 ml 1 M MgSO_4_, H_2_O to 1 L, and sterilized by autoclaving. Bleaching solution was prepared with 0.5 ml 5 M NaOH, 1 ml bleach, and 3.5 ml H_2_O.

### Compound synthesis and characterization

The PROTACs designed were synthesized by Grit Science Inc. and later characterized by nuclear magnetic resonance (NMR) spectroscopy and liquid chromatography-mass spectrometry (LC-MS) to ascertain their purity. More details as described in the supporting information.

### Stable cell line establishment and gene knockdown

The 293T and U251 cell lines were cultured in DMEM medium (Thermo Scientific) supplemented with 10% fetal bovine serum (ExCell Bio) and 1% penicillin-streptomycin (Thermo Scientific). The SH-SY5Y cell line was maintained in DMEM/F-12 medium (Thermo Scientific) containing 15% fetal bovine serum (ExCell Bio) and 1% penicillin-streptomycin (Thermo Scientific). All the cell lines were validated and tested for *mycoplasma*.

Stable cell lines overexpressing α-syn^WT^ or α-syn^A53T^ in 293T, U251, and SH-SY5Y cells were generated using lentiviral transduction. For lentivirus production, 293T cells were co-transfected with the packaging vector psPAX2, the envelope vector pMD2.G, and the transfer vector carrying α-syn^WT/A53T^ (with or without GFP fusion) using polyethyleneimine on the first afternoon. The culture medium was replaced with fresh medium the following morning. After 48 h of incubation at 37 °C, the virus-containing supernatants were collected and filtered through 0.45 μm filters. Before infection, 293T, U251, and SH-SY5Y cells were seeded in 6-well plates and incubated overnight at 37 °C. The medium was then replaced with a 1:1 mixture of fresh medium and virus supernatant. To enhance infection efficiency, 8 μg/ml polybrene was added. Stable cell lines overexpressing α-syn^WT/A53T^ were selected using 1 μg/ml puromycin.

For gene knockdown, shRNA was employed. Lentiviral supernatants for shRNA targeting UBR1, UBR2, UBR4, and UBR5 were prepared and used to infect cells following the same protocol as described for generating stable cell lines overexpressing α-syn^WT/A53T^. The shRNA sequences targeting UBR1, UBR2, UBR4, and UBR5 are provided in Table S1.

### Western blot

After removing the medium, the cells were washed with 1 × PBS buffer and lysed using RIPA buffer (supplemented with 100 × complete protease inhibitor cocktail immediately before use) on ice. The cell lysates were then sonicated at 100 W for 1 min on ice. A 10 μl aliquot of the protein samples was used to determine protein concentration using the BCA protein assay kit (Thermo Scientific). The remaining protein samples were mixed with 5 × loading buffer and denatured by heating at 100°C for 8 min. The protein lysates were subsequently separated by SDS-PAGE and transferred onto polyvinylidene fluoride (PVDF) membranes (Millipore). The membranes were blocked with 5% nonfat milk for 1 h at room temperature, followed by incubation with appropriately diluted primary antibodies in 5% nonfat milk for 1 h at room temperature. After washing three times with 1 × TBST buffer for 5 min each, the membranes were incubated with secondary antibodies for 1 h at room temperature. The membranes were then washed again three times with 1 × TBST buffer for 5 min each. Finally, the membranes were exposed to ECL reagent (Millipore), and protein bands were visualized using the Tanon-5200 Automatic Chemiluminescence Imaging Analysis System (Tanon) and quantitatively analyzed by ImageJ.

### Cell viability determination

A 100 μl suspension of SH-SY5Y cells (1 × 10^4^ cells) was seeded into each well of 96-well plates. On the following day, various concentrations of the compounds were added and incubated for 48 h. After incubation, 10 μl of CCK-8 solution (Yeasen) was added to each well, and the plates were placed in a dark incubator at 37 °C for 3 h. Cell viability was assessed by measuring the absorbance at 450 nm for each well.

### *Candida elegans* strains and their maintenance

*The C*. *elegans* strains N2, NL5901 were obtained from the *Caenorhabditis* Genetics Center. Transgenic strains UM0009 (P_*dat-1*_::GFP) (49), UM0020 (P_*unc-119*_::YFP, P_*unc-119*_::sid-1, P_*dat- 1*_::GFP, P_*aex-3*_:: HASN^A53T^) (50) were established by prof. Garry’s laboratory (Faculty of Health Sciences, Macau University). All the *C*. *elegans* were maintained in the plate filled with Nematode Growth Medium (NGM) agar and coated with *Escherichia coli* OP50 at 16 °C or 20 °C (60). M9 Buffer was used to wash worms and their eggs. While bleaching solution was freshly prepared and used to treat the gravid adult worms to isolate their eggs and synchronize the worms.

### Thrashing assay for *C*. *elegans*

After synchronization with a bleaching solution and allowing the eggs to hatch to the L1 stage, the worms were transferred to fresh NGM plates and fed with OP50 bacteria mixed with varying concentrations of the compounds. The plates were incubated at 20°C in a dark incubator. Day-1 or day-5 adult worms were then selected and placed in a drop of M9 buffer on a glass slide for observation. Following a 30-s equilibration period at room temperature, the number of body bends was counted visually under a stereomicroscope (Nikon SMZ 1270) in 30 s. A bend was defined as the worm's head crossing the midline of its body. For each group, at least 30 worms were assayed.

### Basal slowing response assay for *C*. *elegans*

For the assay plates, a thin layer of freshly prepared OP50 bacteria was evenly spread onto NGM plates. *C*. *elegans* were synchronized and cultured on NGM plates containing OP50 bacteria, either alone or mixed with the compounds, at 20 °C until they developed into day-5 adults. The adult worms were subsequently rinsed with M9 buffer and moved to a clear section of the assay plates. After a 5-minute adaptation period, the number of body bends was counted in 20 s. The N2 wild-type strain was used as the negative control. For each group, at least 30 worms were assayed.

### Microscopy and imaging

For imaging preparation of SH-SY5Y cells, cells were seeded in a 24-well plate, each well containing a circular coverslip. Following compound treatment, the cells were washed with precooled 1 × PBS buffer and fixed with 4% paraformaldehyde (Beyotime, Cat. No.: P0099) for 15 min. The cells were then washed again with precooled 1 × PBS buffer before and after a 10-min treatment with 0.1% Triton X-100 (Sigma). Finally, the circular coverslips were carefully removed from the 24-well plate and mounted onto glass slides with a drop of antifade mounting medium containing DAPI (MCE, Cat. No.: HY-K1047). The slides were sealed using nail polish.

For *C*. *elegans* imaging preparation, a circular line of grease was drawn on a glass slide, and a drop of M9 buffer containing 30 mM levamisole was placed in the center. Worms for observation were transferred into the M9 buffer with levamisole. Once the worms were paralyzed, a coverslip was gently placed over the sample and pressed slowly to avoid damaging the worms.

Imaging and observation were conducted using a Zeiss confocal microscope (LSM980) to visualize GFP and α-synuclein aggregates (α-syn fused with GFP) in SH-SY5Y cells, as well as dopaminergic neurons labeled by GFP and α-synuclein aggregates fused with YFP in the muscle cells of *C*. *elegans*. A 20 × or 40 × objective lens was used for imaging. The 488 nm laser was utilized to excite GFP and YFP, while the 405 nm laser was employed for DAPI excitation.

### Statistical analysis

Data analysis was conducted using GraphPad Prism 9 software, and results are presented as mean ± SEM. For comparisons involving multiple independent groups based on a single factor, one-way ANOVA followed by Tukey's multiple comparisons test was applied. For comparisons involving two nominal predictor variables on a continuous outcome variable, two-way ANOVA followed by Sidak's multiple comparisons test was used. A *p*-value < 0.05 was considered statistically significant, while "ns" indicates no significance (*p* > 0.05).

## Data availability

All experimental procedures and datasets presented in this manuscript or supplementary materials can be obtained upon request from either the first author or the corresponding author.

## Supporting information

This article contains supporting information.

## Conflict of interest

The authors declare that they have no conflicts of interest with the contents of this article.
