# Supplementary material for: Targeted degradation of α-synuclein by arginine-based PROTACs
Source: J Biol Chem. 2025 Jul 2;301(8):110449. doi: 10.1016/j.jbc.2025.110449 (PMC12312037; doi:10.1016/j.jbc.2025.110449)

**Targeted degradation of** **α-synuclein by arginine–based PROTACs**

Linjing Shen^1^, Jianchao Zhang^1^, Zhaoran Wang^1^, Yaxuan Liu^1^, Shengjin Cui^2*^, Hai Rao^1,3*^

^1^ Department of Biochemistry, SUSTech Homeostatic Medicine Institute, School of Medicine, Southern University of Science and Technology, Shenzhen 518055, China

^2^ Clinical Laboratory, The University of Hong Kong-Shenzhen Hospital, Shenzhen, China.

^3^ Key University Laboratory of Metabolism and Health of Guangdong, Southern University of Science and Technology, Shenzhen 518055, China

*To whom correspondence should be addressed: Hai Rao, Department of Biochemistry, School of Medicine, Southern University of Science and Technology, Shenzhen 518055, China. Phone: +86 (0755)88018248, Email: [raoh@sustech.edu.cn](mailto:raoh@sustech.edu.cn); Shengjin Cui, Clinical Laboratory, The University of Hong Kong-Shenzhen Hospital, Shenzhen, China, E-mail: cuisj@hku-szh.org

**List of materials:**

Table S1. Target sequences for shRNA targeting UBR1, UBR2, UBR4 and UBR5 S2

Figure S1. The effect of the proteasome inhibitor on α-syn reduction by immunoblotting in U251/α-syn^A53T^ cells S2

Figure S2. Immunoblot analysis of U251/α-syn^WT^ cells S3

Figure S3. Fluorescence microscope images of mammalian cells S3

Figure S4. Thrashing assay for day-1 adults of C. elegans S4

PROTAC synthesis and characterization S5

1. Synthesis procedure of Arg-PEG1-T^α-syn^ S5

2. NMR and LC-MS characterization of the PROTACs S6

2.1 NMR of PEG1-T^α-syn^ S6

2.2 LC/MS of PEG1-T^α-syn^ S7

2.3 NMR of Arg-PEG1-T^α-syn^ S8

2.4 LC/MS of Arg-PEG1-T^α-syn^ S9

2.5 NMR of Arg-PEG3-T^α-syn^ S10

2.6 LC/MS of Arg-PEG3-T^α-syn^ S11

2.7 NMR of Arg-PEG4-T^α-syn^ S12

2.8 LC/MS of Arg-PEG4-T^α-syn^ S13

**Table S1.** Target sequences for shRNA targeting UBR1, UBR2, UBR4 and UBR5.

| **Name** | **Target Sequences** |
| --- | --- |
| shUBR1 #1 | GCGTTGAGTCTTCGATTAAAT |
| shUBR1 #2 | CCAAGAGACTAATCAGATGTT |
| shUBR2 #1 | CCTCCTTACCTTGATGACTAT |
| shUBR2 #2 | GCCGGAATGTGGAGAAGAAAT |
| shUBR4 #1 | CCACATACATTGTTCGGGAAA |
| shUBR4 #2 | CCACCATCAAAGACTTACATT |
| shUBR5 #1 | TTGGAACAGGCTACTATTAAA |
| shUBR5 #2 | GCTGTAGATTTCAACTTAGAT |


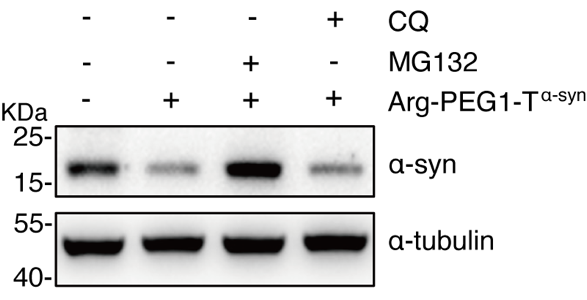


**Figure S1. The effect of the proteasome inhibitor on α-syn reduction by immunoblotting in U251/α-syn^A53T^ cells.**

The U251/α-synA^53T^ cells were treated for 24 h with DMSO, 1 μM Arg-PEG1-T^α-syn^, or Arg-PEG1-T^α-syn^ combined with either 2 μM MG132 (proteasome inhibitor) or 2 μM CQ (autophagy inhibitor).


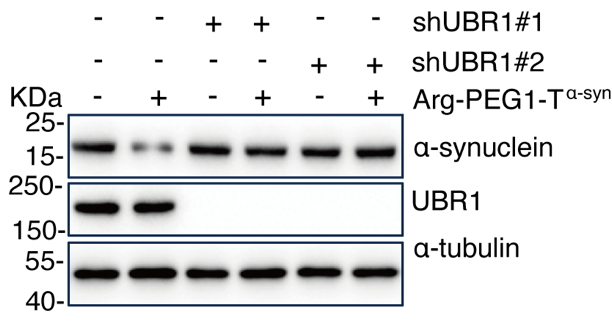


**Figure S2. Immunoblot analysis of U251/α-syn^WT^ cells.**

The U251/α-syn^WT^ cells stably expressing shRNA targeting UBR1 were treated with either DMSO or 1 μM Arg-PEG1-T^α-syn^ for 48 hours.


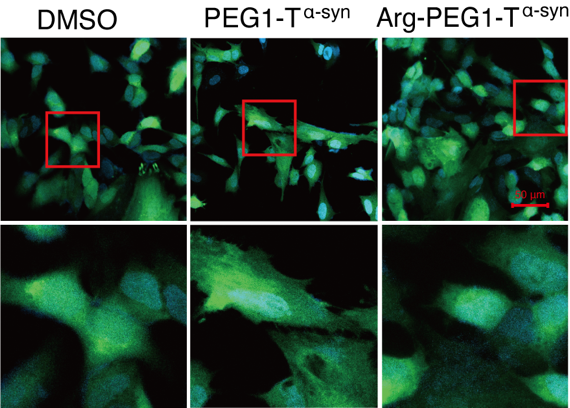


**Figure S3. Fluorescence microscope images of mammalian cells.**

SH-SY5Y cells stably co-expressing α-syn^A53T^ and GFP separately through lentivirus infection were treated with 5 μM of the respective compounds.


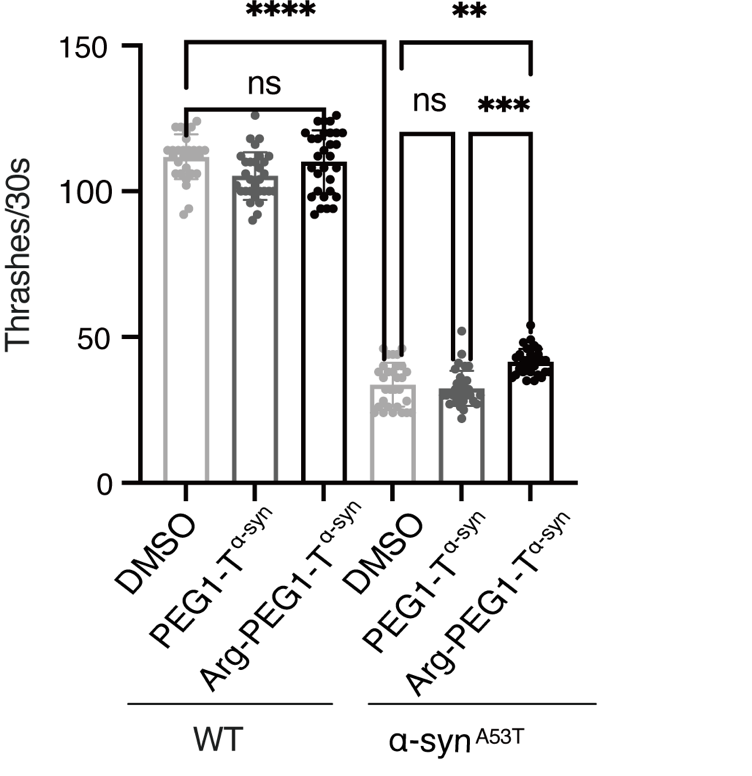


**Figure S4. Thrashing assay for day-1 adults of *C. elegans*.**

The WT strain and the α-syn^A53T^ strain were used for this test and treated with 5 μM of compounds. One-way ANOVA with Tukey's multiple comparisons test was used. F=824.3, *p*<0.0001. ** *p*<0.01, *** *p*<0.001, **** *p*<0.0001, ns: *p*>0.05. All individual data points super-imposed on the bar graph were independent biological replicates.

**PROTAC** **synthesis and characterization**

1. **Synthesis procedure of** **Arg-PEG1-T^α-syn^:**


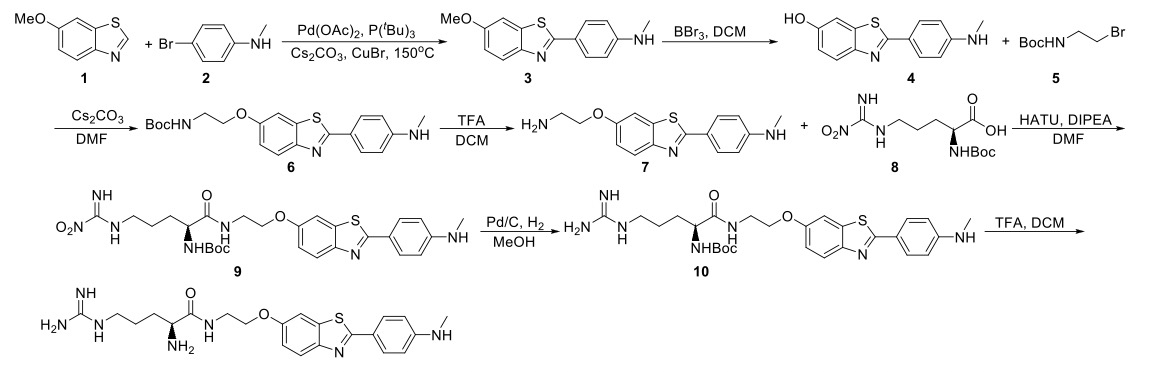


For the synthesis of Arg-PEG₁-T^α-syn^, we first prepared the BocHN-PEG₁-Tα-syn intermediate, containing a single PEG unit. This intermediate was then conjugated with arginine Arg to yield the target PROTAC molecule. The syntheses of Arg-PEG₃-T^α-syn^ and Arg-PEG₄-T^α-syn^ followed an analogous procedure to that of Arg-PEG₁-T^α-syn^. PEG₁-T^α-syn^ is an intermediate product in the synthesis process.

1. **NMR and LC-MS characterization of the PROTACs**

The successful formation of the desired compounds was verified by nuclear magnetic resonance (NMR) spectroscopy and liquid chromatography-mass spectrometry (LC-MS).

**2.1 NMR of PEG1-T^α-syn^**


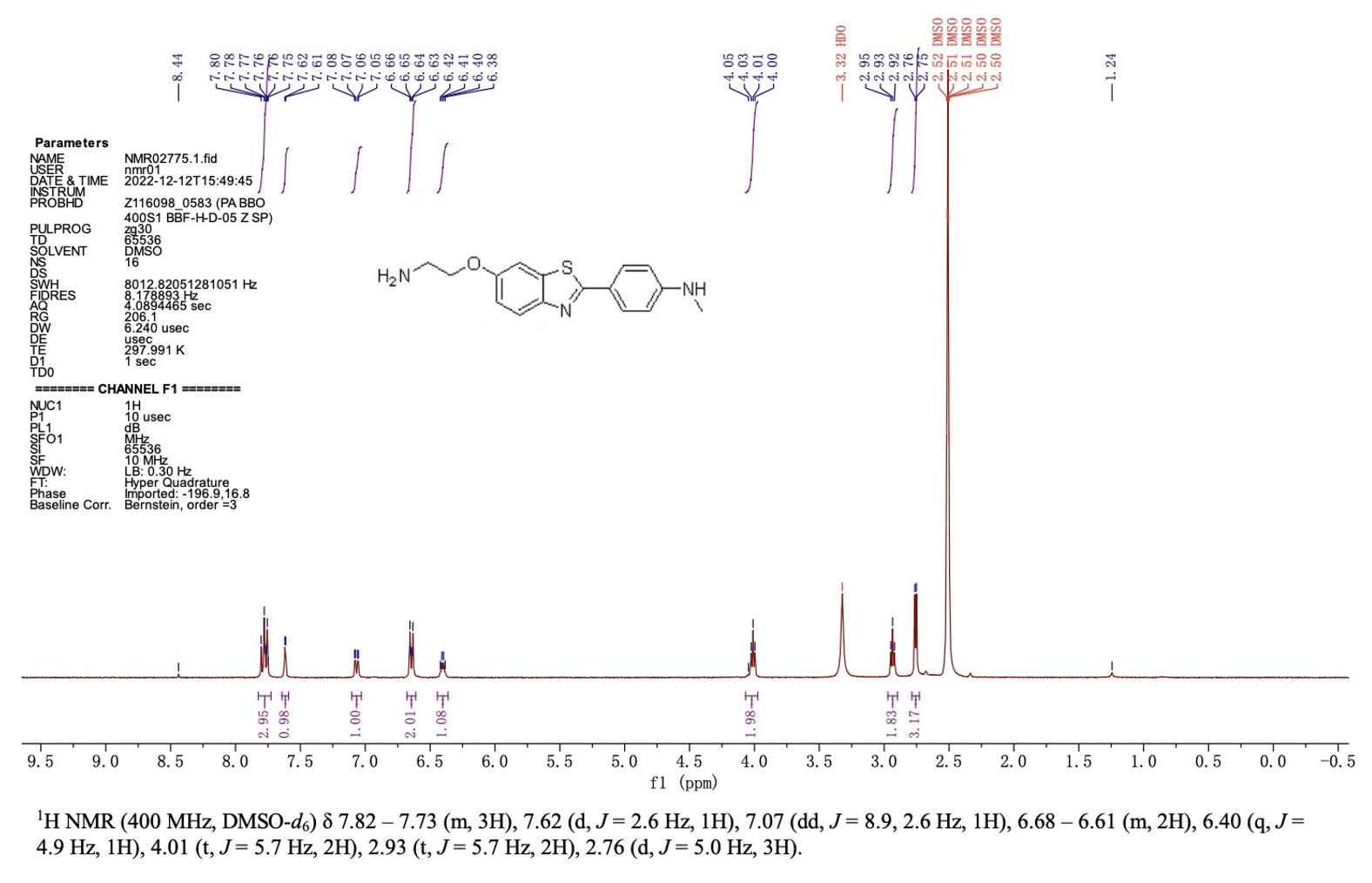


**2.2 LC/MS of PEG1-T^α-syn^**


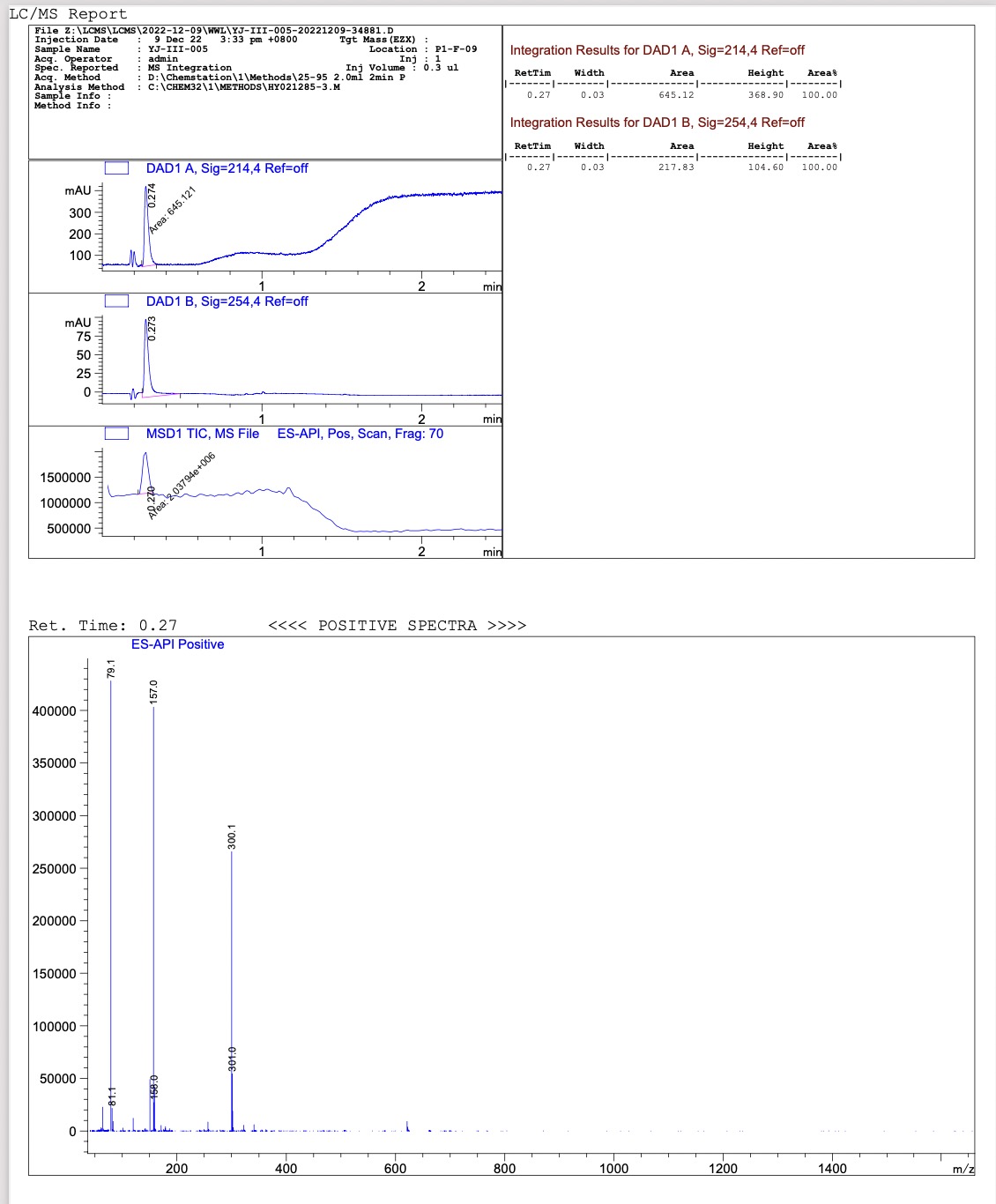


**2.3 NMR of** **Arg-PEG1-T^α-syn^**


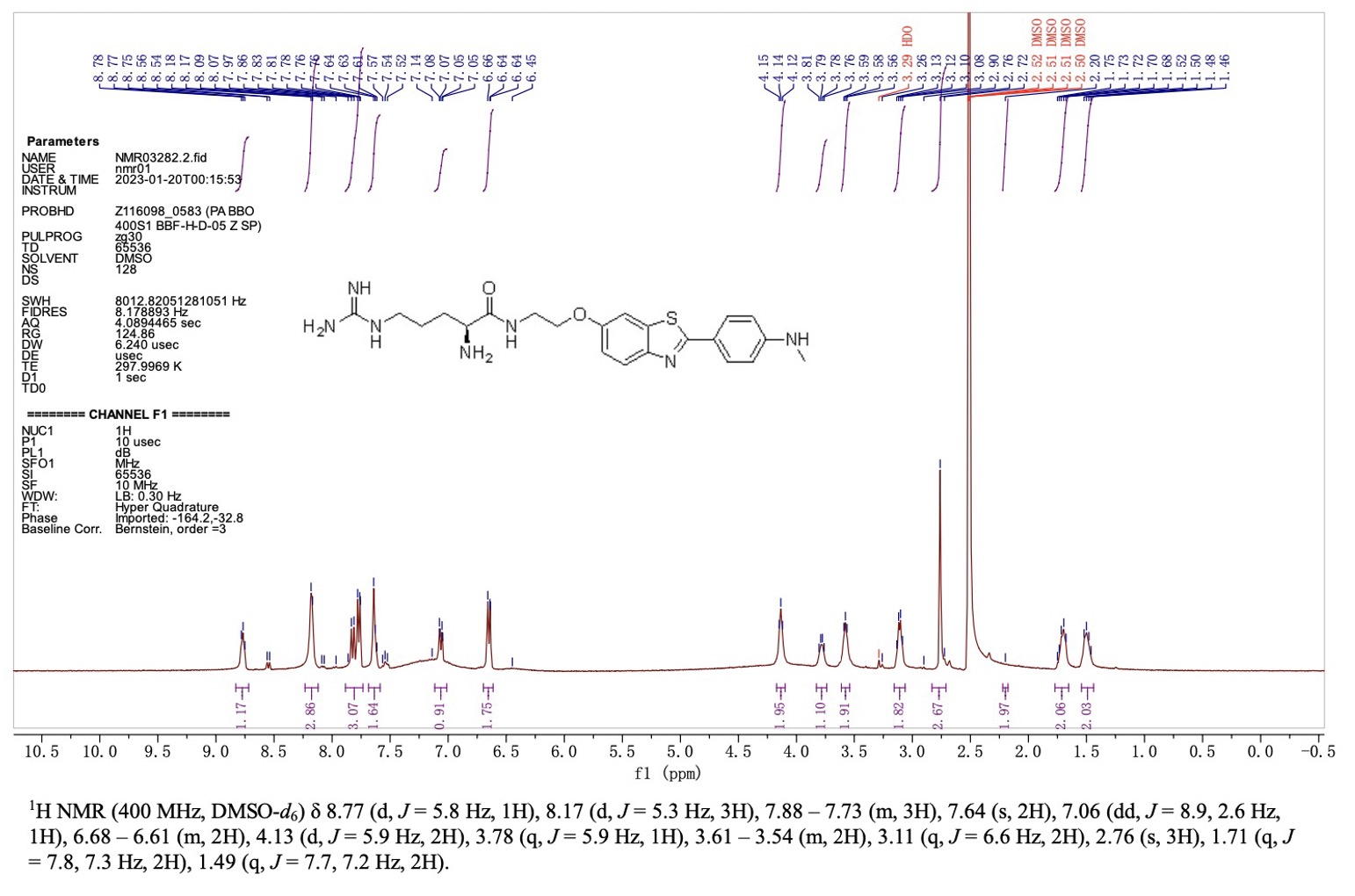


**2.4 LC/MS of Arg-PEG1-T^α-syn^**


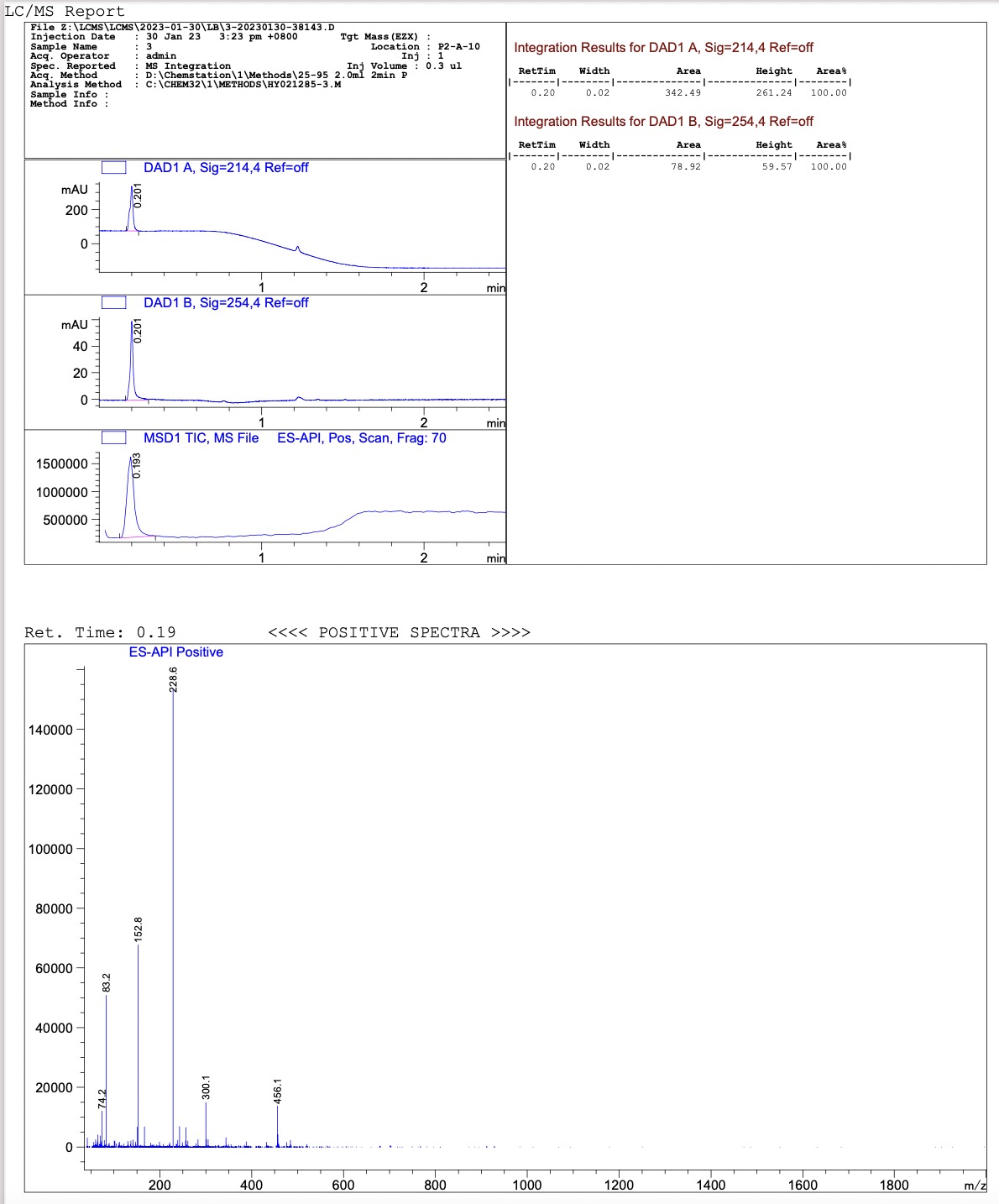


**2.5 NMR of Arg-PEG3-T^α-syn^**

**^
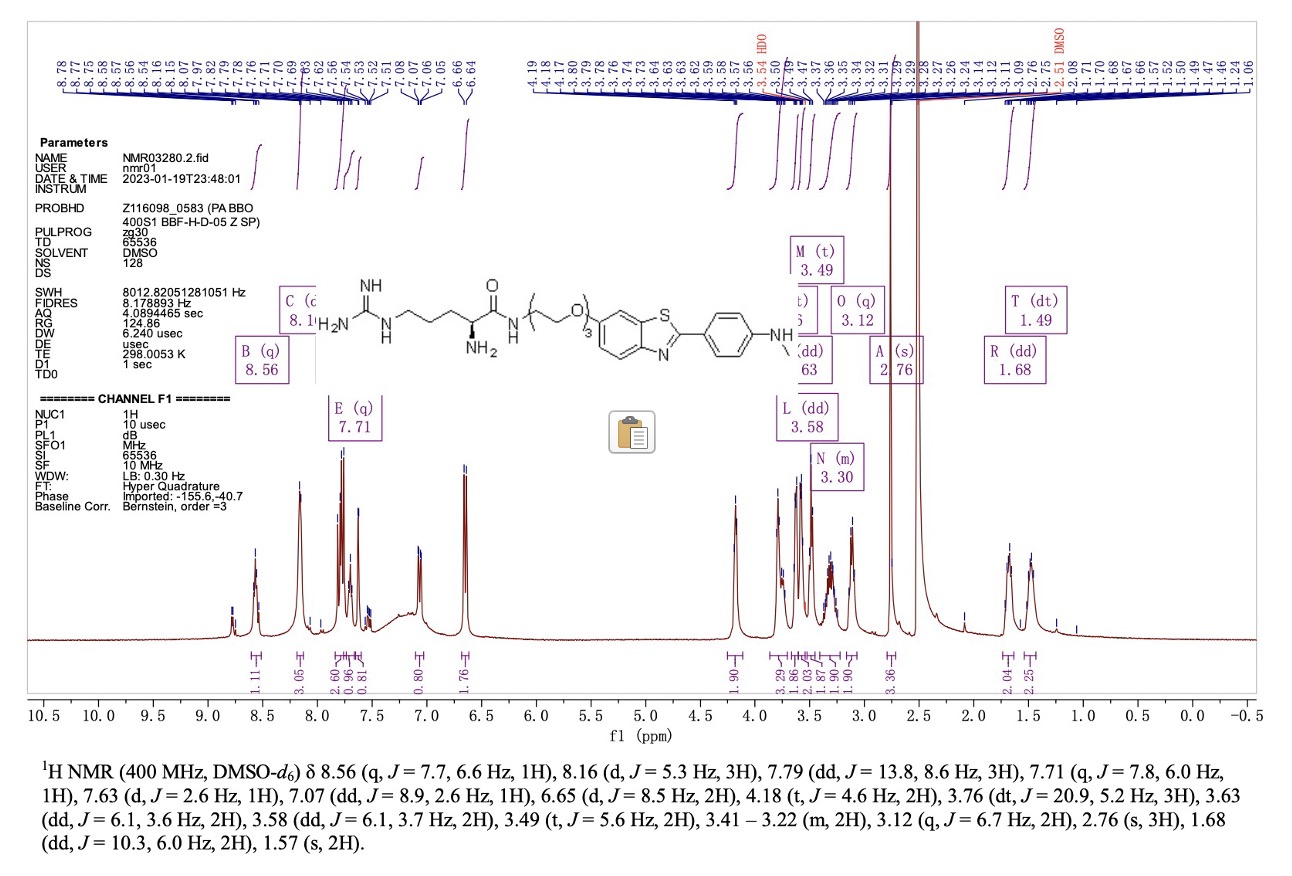
^**

**2.6 LC/MS of Arg-PEG3-T^α-syn^**


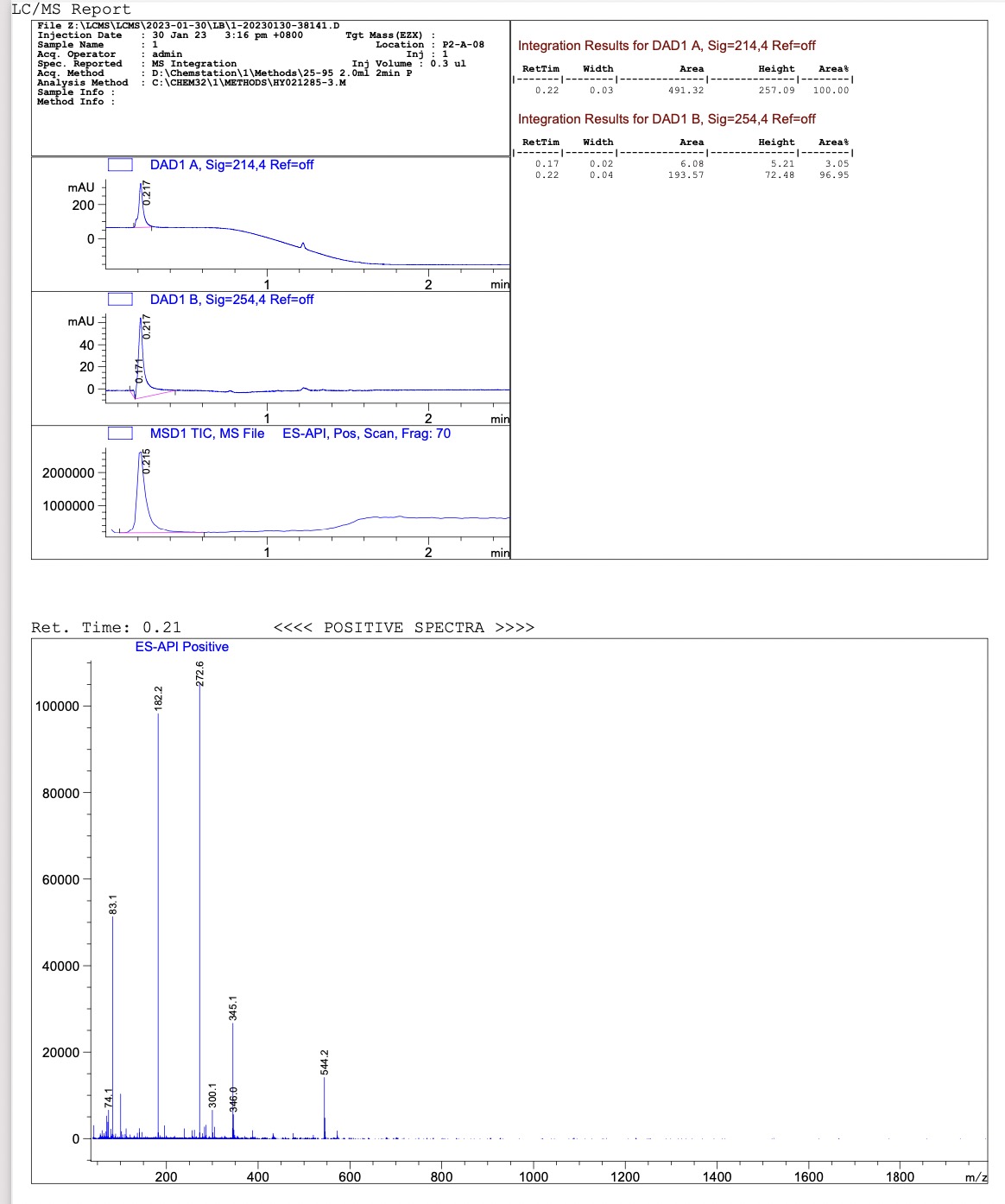


**2.7 NMR of Arg-PEG4-T^α-syn^**

**^
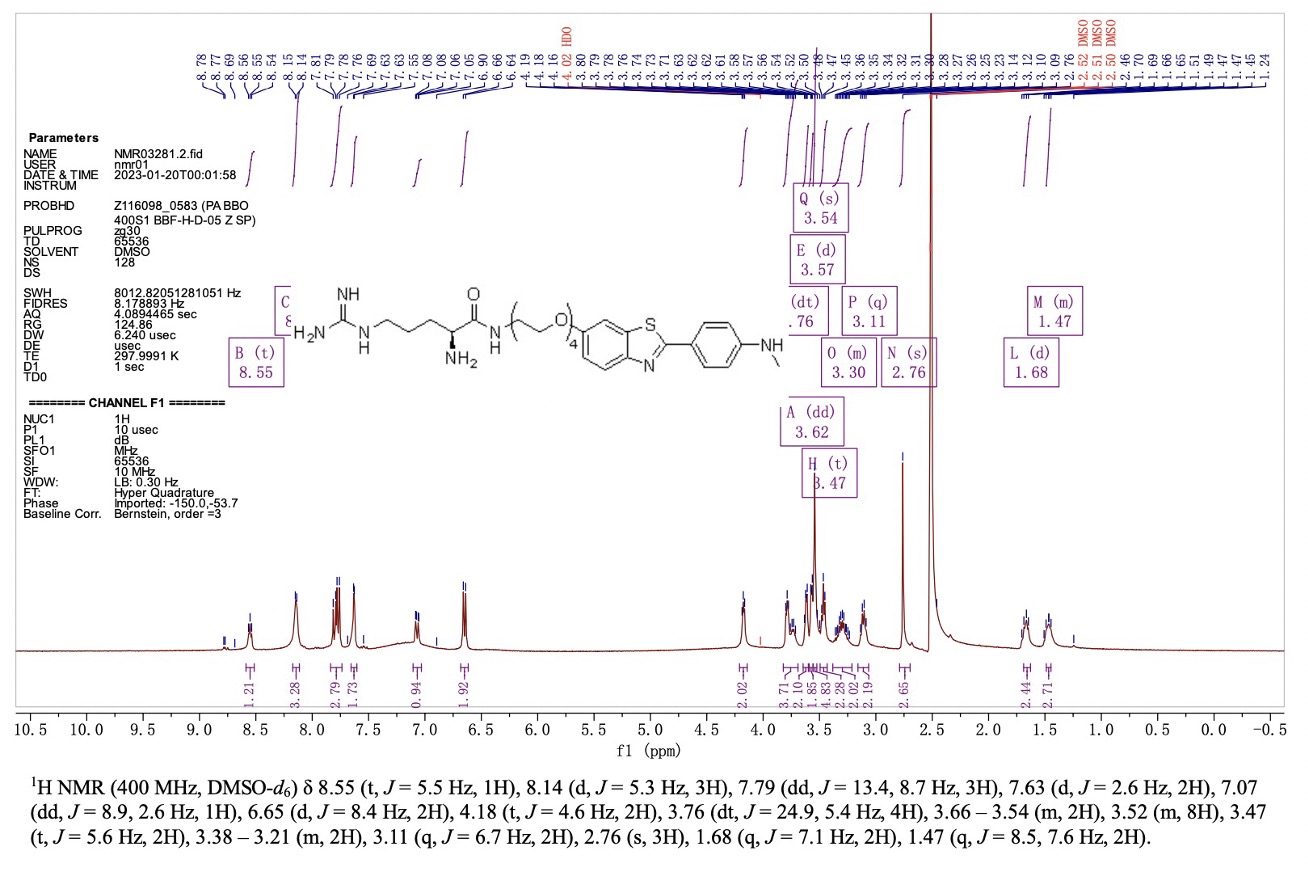
^**

**2.8 LC/MS of Arg-PEG4-T^α-syn^**


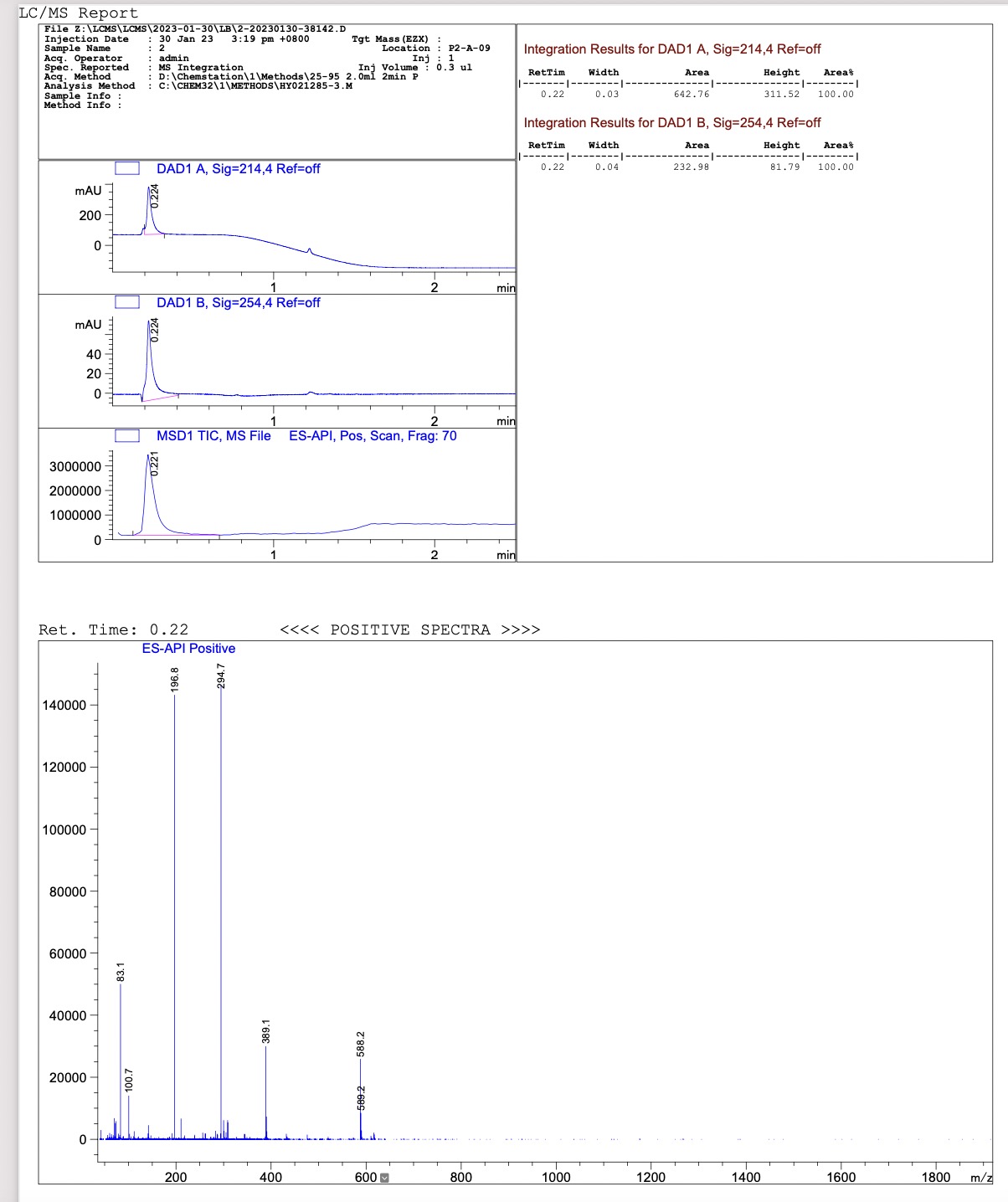

Supplement: Supplementary Data [file mmc1.docx]
